# Supplementary material for: Mechanisms governing the pioneering and redistribution capabilities of the non-classical pioneer PU.1
Source: Nat Commun. 2020 Jan 21;11:402. doi: 10.1038/s41467-019-13960-2 (PMC6972792; doi:10.1038/s41467-019-13960-2)
Supplement: Supplementary file 7 — Source data [file 41467_2019_13960_MOESM7_ESM.zip › Source_Data/Figure6/Figure6B_MotifScanOutput/ETS1_mutPU.1/homerResults.html]

/misc/data/analysis/project\_PU1/CTV1/motifs/basic/peaksETS1\_DSG\_PU1mut\_merged/final - Homer de novo Motif Results


# Homer *de novo* Motif Results (/misc/data/analysis/project\_PU1/CTV1/motifs/basic/peaksETS1\_DSG\_PU1mut\_merged/final)

Known Motif Enrichment Results  
Gene Ontology Enrichment Results  
If Homer is having trouble matching a motif to a known motif, try copy/pasting the matrix file into
STAMP  
More information on motif finding results: HOMER
| Description of Results
| Tips
  
Total target sequences = 13485  
Total background sequences = 35094  
\* - possible false positive  

|  |  |  |  |  |  |  |  |  |
| --- | --- | --- | --- | --- | --- | --- | --- | --- |
| Rank | Motif | P-value | log P-pvalue | % of Targets | % of Background | STD(Bg STD) | Best Match/Details | Motif File |
| 1 | C T G A T A G C T G A C C A T G T A C G T G C A G C T A T C A G A G C T T A C G T C A G | 1e-3059 | -7.044e+03 | 75.01% | 17.73% | 43.0bp (63.8bp) | Fli1(ETS)/CD8-FLI-ChIP-Seq(GSE20898)/Homer(0.979) More Information \| Similar Motifs Found | motif file (matrix) |
| 2 | A G T C G A C T G C T A T C G A T C G A A G T C A G T C C G T A A G T C G C T A C T A G | 1e-1163 | -2.678e+03 | 41.76% | 10.99% | 47.7bp (61.2bp) | RUNX1(Runt)/Jurkat-RUNX1-ChIP-Seq(GSE29180)/Homer(0.980) More Information \| Similar Motifs Found | motif file (matrix) |
| 3 | G T C A A G T C C G T A A G C T A C G T A C G T A G T C A G T C | 1e-700 | -1.614e+03 | 28.93% | 8.00% | 53.8bp (62.9bp) | Eip74EF/dmmpmm(SeSiMCMC)/fly(0.764) More Information \| Similar Motifs Found | motif file (matrix) |
| 4 | C T G A T A G C T G C A C A T G A T C G C T G A A G C T C T A G C G A T A C T G C T A G A T C G | 1e-459 | -1.058e+03 | 15.39% | 3.12% | 49.9bp (59.2bp) | ETS:RUNX(ETS,Runt)/Jurkat-RUNX1-ChIP-Seq(GSE17954)/Homer(0.902) More Information \| Similar Motifs Found | motif file (matrix) |
| 5 | T G C A A C G T A C T G G C T A C T A G A C G T T A G C C G T A A G C T | 1e-246 | -5.683e+02 | 11.23% | 3.08% | 53.0bp (59.0bp) | JDP2/MA0655.1/Jaspar(0.978) More Information \| Similar Motifs Found | motif file (matrix) |
| 6 | T A C G C A T G C A T G T A C G C T G A T C G A T A C G A G C T A C T G A C T G A C G T C A G T C T G A | 1e-213 | -4.916e+02 | 4.43% | 0.34% | 44.1bp (60.5bp) | ETS:RUNX(ETS,Runt)/Jurkat-RUNX1-ChIP-Seq(GSE17954)/Homer(0.837) More Information \| Similar Motifs Found | motif file (matrix) |
| 7 | A C G T C G A T T G C A A G T C A C T G A C G T G A T C | 1e-205 | -4.733e+02 | 14.42% | 5.56% | 56.7bp (68.8bp) | YAP3/MA0416.1/Jaspar(0.831) More Information \| Similar Motifs Found | motif file (matrix) |
| 8 | G T A C G A T C C A G T A G T C A T G C G A T C G T A C A G T C C T G A A T G C G T C A | 1e-196 | -4.535e+02 | 27.97% | 15.69% | 52.6bp (60.2bp) | EGR1/MA0162.2/Jaspar(0.911) More Information \| Similar Motifs Found | motif file (matrix) |
| 9 | C T A G G C A T A G C T A T G C G A C T G C A T C G T A C G A T A G T C C G A T A T C G | 1e-187 | -4.328e+02 | 15.20% | 6.38% | 52.4bp (62.2bp) | GATA3(Zf)/iTreg-Gata3-ChIP-Seq(GSE20898)/Homer(0.975) More Information \| Similar Motifs Found | motif file (matrix) |
| 10 | A G T C G A C T A C G T C A G T T A C G G C T A C G A T A T C G C G A T C A T G C A T G A G C T | 1e-170 | -3.929e+02 | 5.53% | 1.01% | 44.0bp (58.0bp) | TCFL2(HMG)/K562-TCF7L2-ChIP-Seq(GSE29196)/Homer(0.821) More Information \| Similar Motifs Found | motif file (matrix) |
| 11 | C T A G A G T C C A G T A C T G G C A T G A C T C G T A C T G A | 1e-150 | -3.461e+02 | 64.95% | 51.86% | 56.4bp (64.3bp) | BMYB(HTH)/Hela-BMYB-ChIP-Seq(GSE27030)/Homer(0.835) More Information \| Similar Motifs Found | motif file (matrix) |
| 12 | G T C A G T A C A G C T T C G A A G T C T C G A G C T A G A C T G A C T G A T C A G T C A G T C | 1e-149 | -3.447e+02 | 4.31% | 0.66% | 48.0bp (68.0bp) | GFY(?)/Promoter/Homer(0.971) More Information \| Similar Motifs Found | motif file (matrix) |
| 13 | C G T A C G T A A T G C A G T C A C T G G C T A C G T A C T G A | 1e-136 | -3.154e+02 | 20.39% | 11.37% | 57.3bp (66.9bp) | MYB73(MYB)/col-MYB73-DAP-Seq(GSE60143)/Homer(0.870) More Information \| Similar Motifs Found | motif file (matrix) |
| 14 | A C T G A C T G A T C G T A C G T C A G C T A G A C T G | 1e-133 | -3.078e+02 | 38.84% | 27.20% | 54.6bp (62.1bp) | ZNF740/MA0753.1/Jaspar(0.900) More Information \| Similar Motifs Found | motif file (matrix) |
| 15 | A G C T T A C G C T A G A G C T A G C T C A T G G T A C | 1e-129 | -2.979e+02 | 32.97% | 22.13% | 57.4bp (61.1bp) | Dux/MA0611.1/Jaspar(0.766) More Information \| Similar Motifs Found | motif file (matrix) |
| 16 | A G T C G A C T A C G T A G T C A G T C A C G T A C G T A G T C G A T C A C T G T A C G | 1e-126 | -2.915e+02 | 7.13% | 2.30% | 51.1bp (69.9bp) | RBM5(Znf)/Homo\_sapiens-RNCMPT00055-PBM/HughesRNA(0.777) More Information \| Similar Motifs Found | motif file (matrix) |
| 17 | A C G T A G C T A G T C G A C T A T C G G A T C C T A G A C T G A G C T A C G T C A T G | 1e-125 | -2.887e+02 | 14.41% | 7.15% | 54.7bp (65.9bp) | RUNX3/MA0684.1/Jaspar(0.757) More Information \| Similar Motifs Found | motif file (matrix) |
| 18 | C A G T A C T G G A C T A C T G C A T G A G C T A C G T C T G A T C G A T G A C A G T C C G T A A T G C | 1e-122 | -2.814e+02 | 7.72% | 2.71% | 51.9bp (60.6bp) | GT-1/MA1020.1/Jaspar(0.774) More Information \| Similar Motifs Found | motif file (matrix) |
| 19 | T G A C G T A C A C T G T A C G C G T A G C T A C T A G C T A G A T G C | 1e-120 | -2.782e+02 | 20.95% | 12.30% | 54.6bp (68.1bp) | Eip74EF/MA0026.1/Jaspar(0.795) More Information \| Similar Motifs Found | motif file (matrix) |
| 20 | C T A G G A T C A T C G T C G A A G T C G T A C C T A G A T G C G T C A C G T A C G T A | 1e-116 | -2.693e+02 | 4.23% | 0.89% | 53.5bp (58.1bp) | EWS:FLI1-fusion(ETS)/SK\_N\_MC-EWS:FLI1-ChIP-Seq(SRA014231)/Homer(0.740) More Information \| Similar Motifs Found | motif file (matrix) |
| 21 | G A T C C G T A C T A G G T A C G A C T A C T G C G A T A G T C C G T A | 1e-110 | -2.536e+02 | 28.05% | 18.61% | 56.1bp (63.1bp) | MyoG(bHLH)/C2C12-MyoG-ChIP-Seq(GSE36024)/Homer(0.921) More Information \| Similar Motifs Found | motif file (matrix) |
| 22 | C G A T C G T A T G A C A G C T A C T G C G A T A G T C C T G A | 1e-98 | -2.275e+02 | 30.17% | 20.93% | 58.8bp (62.5bp) | Tgif2(Homeobox)/mES-Tgif2-ChIP-Seq(GSE55404)/Homer(0.821) More Information \| Similar Motifs Found | motif file (matrix) |
| 23 | G C A T T G C A C A G T T A G C G C T A C G T A T G A C G A T C T C A G A T G C | 1e-98 | -2.268e+02 | 24.29% | 15.85% | 55.7bp (63.6bp) | GAMYB(MYB)/Hordeum vulgare/AthaMap(0.724) More Information \| Similar Motifs Found | motif file (matrix) |
| 24 | C T A G T C A G A G T C G A T C G T A C A G T C C A T G A G T C A G T C A G T C | 1e-95 | -2.193e+02 | 18.96% | 11.55% | 56.5bp (62.9bp) | KLF5/MA0599.1/Jaspar(0.907) More Information \| Similar Motifs Found | motif file (matrix) |
| 25 | C T G A A C T G A G C T A C T G A C T G A C G T G C T A T C A G | 1e-88 | -2.035e+02 | 8.39% | 3.75% | 53.5bp (61.8bp) | ZBTB7C/MA0695.1/Jaspar(0.765) More Information \| Similar Motifs Found | motif file (matrix) |
| 26 | A G T C A C G T A C G T A C G T A T C G C G T A C G A T | 1e-84 | -1.946e+02 | 13.82% | 7.82% | 53.2bp (66.1bp) | pan/dmmpmm(Papatsenko)/fly(0.949) More Information \| Similar Motifs Found | motif file (matrix) |
| 27 | G A C T C G A T G T A C A G T C A G T C T C G A A T C G G T C A C T G A | 1e-83 | -1.932e+02 | 7.65% | 3.34% | 52.7bp (63.7bp) | STAT1/MA0137.3/Jaspar(0.921) More Information \| Similar Motifs Found | motif file (matrix) |
| 28 | C G A T A T C G C G A T C T A G C A T G A T G C C G A T A T C G T G C A A G C T C G A T | 1e-72 | -1.660e+02 | 7.50% | 3.50% | 54.6bp (58.3bp) | CRZ1(MacIsaac)/Yeast(0.795) More Information \| Similar Motifs Found | motif file (matrix) |
| 29 | T C A G C G A T T A G C C G A T A T C G A C G T A G C T A G C T A G T C G A T C C G A T A C T G G A C T T G C A | 1e-68 | -1.574e+02 | 2.56% | 0.56% | 50.6bp (61.0bp) | ETS:E-box(ETS,bHLH)/HPC7-Scl-ChIP-Seq(GSE22178)/Homer(0.829) More Information \| Similar Motifs Found | motif file (matrix) |
| 30 | T A G C G T A C T G A C T G C A A T G C G T C A T G C A C G T A A T G C G A T C C T A G | 1e-66 | -1.540e+02 | 3.70% | 1.17% | 53.5bp (63.8bp) | RBM38(RRM)/Mus\_musculus-RNCMPT00051-PBM/HughesRNA(0.779) More Information \| Similar Motifs Found | motif file (matrix) |
| 31 | G C T A T C G A G T C A T A G C G A T C T C A G T C G A G T C A T C G A T A G C G A T C | 1e-56 | -1.293e+02 | 6.24% | 2.99% | 57.1bp (66.9bp) | PB0035.1\_Irf5\_1/Jaspar(0.862) More Information \| Similar Motifs Found | motif file (matrix) |
| 32 | C G A T C A T G C G T A A G T C T C A G G C A T G A T C G T C A A C G T G A T C | 1e-53 | -1.230e+02 | 6.83% | 3.48% | 55.9bp (58.6bp) | TGA1(bZIP)/colamp-TGA1-DAP-Seq(GSE60143)/Homer(0.934) More Information \| Similar Motifs Found | motif file (matrix) |
| 33 | T G A C C G T A A C T G A C G T C A G T C A T G A T C G C G A T C A T G A C T G A G C T C A G T C A G T | 1e-52 | -1.209e+02 | 2.90% | 0.91% | 51.9bp (62.2bp) | AT1G24250(Orphan)/col-AT1G24250-DAP-Seq(GSE60143)/Homer(0.715) More Information \| Similar Motifs Found | motif file (matrix) |
| 34 | A T G C C G A T A C T G C A G T A C T G T A C G T A G C G A C T A C G T C A G T A C T G G T C A A C G T | 1e-51 | -1.189e+02 | 2.99% | 0.98% | 55.5bp (60.5bp) | RUNX1/MA0002.2/Jaspar(0.723) More Information \| Similar Motifs Found | motif file (matrix) |
| 35 | C A G T T A G C C G T A T C G A C G T A T C G A A T C G T A G C | 1e-49 | -1.145e+02 | 13.88% | 9.14% | 57.3bp (66.2bp) | Sf3b4(RRM)/Danio\_rerio-RNCMPT00224-PBM/HughesRNA(0.898) More Information \| Similar Motifs Found | motif file (matrix) |
| 36 | G T A C T C A G T C A G C A G T A C T G A T G C G T C A A G C T A C G T T A G C A G C T A C T G A C T G A C T G | 1e-49 | -1.136e+02 | 1.91% | 0.43% | 51.5bp (56.7bp) | ZNF143\|STAF(Zf)/CUTLL-ZNF143-ChIP-Seq(GSE29600)/Homer(0.849) More Information \| Similar Motifs Found | motif file (matrix) |
| 37 | C T G A A G T C A C T G A C G T A T G C C G T A A G T C | 1e-39 | -9.066e+01 | 8.38% | 5.10% | 55.8bp (64.0bp) | TGA5/MA1047.1/Jaspar(0.924) More Information \| Similar Motifs Found | motif file (matrix) |
| 38 | T C G A A T C G A T G C A G T C C G T A C T G A A G C T A G T C T C A G T A C G | 1e-38 | -8.932e+01 | 8.29% | 5.05% | 56.5bp (58.8bp) | NFY(CCAAT)/Promoter/Homer(0.903) More Information \| Similar Motifs Found | motif file (matrix) |
| 39 | C A G T T C A G T G A C T C A G A T G C G T C A C A G T A T C G A G T C C A T G A G T C | 1e-38 | -8.889e+01 | 3.25% | 1.35% | 55.6bp (70.6bp) | NRF1(NRF)/MCF7-NRF1-ChIP-Seq(Unpublished)/Homer(0.978) More Information \| Similar Motifs Found | motif file (matrix) |
| 40 | T G C A T A G C A G C T T A G C A G T C G C T A A C G T A C G T C G A T A G T C A G T C A G T C C G T A | 1e-38 | -8.790e+01 | 0.79% | 0.06% | 50.7bp (0.0bp) | Rbpj1(?)/Panc1-Rbpj1-ChIP-Seq(GSE47459)/Homer(0.766) More Information \| Similar Motifs Found | motif file (matrix) |
| 41 | T C A G A G C T A T G C G T C A A G T C C T A G A C G T A T C G T C G A A G T C A C G T | 1e-37 | -8.521e+01 | 3.43% | 1.51% | 54.5bp (59.9bp) | TFEB/MA0692.1/Jaspar(0.965) More Information \| Similar Motifs Found | motif file (matrix) |
| 42 | C G A T A G T C A G C T C T A G G C T A C A T G G T A C | 1e-36 | -8.303e+01 | 33.56% | 27.69% | 57.8bp (63.6bp) | CG14718(RRM)/Drosophila\_melanogaster-RNCMPT00006-PBM/HughesRNA(0.792) More Information \| Similar Motifs Found | motif file (matrix) |
| 43 | C T G A T G C A T C A G T C G A A C G T T C A G C T A G T G A C A C T G A T C G | 1e-33 | -7.685e+01 | 2.74% | 1.12% | 59.1bp (63.9bp) | YY1(Zf)/Promoter/Homer(0.849) More Information \| Similar Motifs Found | motif file (matrix) |
| 44 | T C G A C G T A A G T C A G C T C T A G T G A C T C G A | 1e-31 | -7.316e+01 | 11.55% | 8.04% | 56.6bp (67.3bp) | Rbm42(RRM)/Xenopus\_tropicalis-RNCMPT00282-PBM/HughesRNA(0.801) More Information \| Similar Motifs Found | motif file (matrix) |
| 45 | A G T C A G C T A G T C A G T C A C G T A G T C A G T C | 1e-31 | -7.305e+01 | 13.96% | 10.11% | 55.4bp (64.3bp) | SRSF1(RRM)/Homo\_sapiens-RNCMPT00163-PBM/HughesRNA(0.948) More Information \| Similar Motifs Found | motif file (matrix) |
| 46 | A C G T A G T C C G A T A C T G G T A C C A T G A C T G A C G T A G C T A G C T C A G T C A T G C G A T A T C G | 1e-29 | -6.835e+01 | 0.54% | 0.03% | 52.2bp (41.8bp) | RUNX3/MA0684.1/Jaspar(0.757) More Information \| Similar Motifs Found | motif file (matrix) |
| 47 | G T A C A G T C C G A T A C T G A G T C A C G T A C T G C G A T A C T G | 1e-28 | -6.601e+01 | 4.92% | 2.80% | 52.2bp (63.0bp) | Unknown-ESC-element(?)/mES-Nanog-ChIP-Seq(GSE11724)/Homer(0.942) More Information \| Similar Motifs Found | motif file (matrix) |
| 48 | T A C G A G T C T G A C T G C A A T G C A G C T C T G A T A C G C T G A C A T G T A C G A T C G G T A C C T A G | 1e-24 | -5.610e+01 | 2.17% | 0.93% | 59.8bp (63.4bp) | BORIS(Zf)/K562-CTCFL-ChIP-Seq(GSE32465)/Homer(0.851) More Information \| Similar Motifs Found | motif file (matrix) |
| 49 | T G A C C A T G C G A T G C A T C T G A T G A C G A T C G C T A C A G T C T G A T A C G | 1e-21 | -4.898e+01 | 2.44% | 1.19% | 55.1bp (61.8bp) | RFX4/MA0799.1/Jaspar(0.745) More Information \| Similar Motifs Found | motif file (matrix) |
| 50 | C A G T A C G T G T C A A C T G C G A T G A T C C T A G G A T C | 1e-19 | -4.421e+01 | 2.75% | 1.47% | 56.8bp (63.0bp) | Rbm42(RRM)/Xenopus\_tropicalis-RNCMPT00282-PBM/HughesRNA(0.760) More Information \| Similar Motifs Found | motif file (matrix) |
| 51 | T C G A A T C G T A G C C G T A T C A G T A G C G T A C G T C A T G C A A C G T T A G C T C G A | 1e-18 | -4.153e+01 | 2.34% | 1.20% | 58.1bp (57.1bp) | POL004.1\_CCAAT-box/Jaspar(0.807) More Information \| Similar Motifs Found | motif file (matrix) |
| 52 | C A T G C G T A C G A T C A T G T G A C A G T C G C T A T A C G C T G A C A T G G C T A C A G T G C T A T C G A | 1e-17 | -3.987e+01 | 0.52% | 0.09% | 55.0bp (48.3bp) | Gata1/MA0035.3/Jaspar(0.696) More Information \| Similar Motifs Found | motif file (matrix) |
| 53 | C T G A T A C G G T C A A G C T A C G T G C A T G A C T A C G T G C T A A C G T A G T C C G A T A T G C G T A C | 1e-17 | -3.985e+01 | 0.58% | 0.12% | 53.7bp (44.2bp) | GATA2/MA0036.2/Jaspar(0.747) More Information \| Similar Motifs Found | motif file (matrix) |
| 54 | C G T A A G C T A C T G A T G C C G A T G T C A A C G T G A T C G C A T T C A G | 1e-16 | -3.910e+01 | 1.50% | 0.65% | 54.6bp (55.8bp) | FZF1/MA0298.1/Jaspar(0.668) More Information \| Similar Motifs Found | motif file (matrix) |
| 55 | C G T A A G T C G T C A A C T G A C T G C G T A A C G T A C T G C G T A | 1e-16 | -3.877e+01 | 0.85% | 0.26% | 51.8bp (58.0bp) | G3BP2(RRM)/Homo\_sapiens-RNCMPT00021-PBM/HughesRNA(0.811) More Information \| Similar Motifs Found | motif file (matrix) |
| 56 | T C A G C G T A G C A T T C G A C T G A T C A G C T G A C A G T A T G C G C T A T G C A T C G A C T G A T C A G | 1e-15 | -3.569e+01 | 0.40% | 0.05% | 50.2bp (63.3bp) | pan/dmmpmm(Bergman)/fly(0.827) More Information \| Similar Motifs Found | motif file (matrix) |
| 57 | G C A T C T G A G T C A A G C T G A T C A G C T G T A C A T C G G A T C C A T G | 1e-15 | -3.507e+01 | 3.16% | 1.91% | 57.0bp (61.8bp) | GFX(?)/Promoter/Homer(0.740) More Information \| Similar Motifs Found | motif file (matrix) |
| 58 | T G A C C A G T T G A C A C G T G T A C A C T G T G A C A T C G T G C A A T C G C T G A | 1e-14 | -3.451e+01 | 0.76% | 0.23% | 57.3bp (60.4bp) | GFX(?)/Promoter/Homer(0.961) More Information \| Similar Motifs Found | motif file (matrix) |
| 59 | C A T G C T A G A C G T A G C T C A T G A G T C A G T C G T C A C A G T C T A G C T A G T G A C T C G A T C G A | 1e-13 | -3.004e+01 | 1.22% | 0.54% | 53.2bp (60.6bp) | Rfx1(HTH)/NPC-H3K4me1-ChIP-Seq(GSE16256)/Homer(0.984) More Information \| Similar Motifs Found | motif file (matrix) |
| 60 | T G A C G C A T A C T G C G T A A C G T A C T G A C G T A T C G A C T G A G T C G A C T T A C G | 1e-12 | -2.927e+01 | 0.28% | 0.03% | 50.1bp (41.3bp) | MET32/MA0334.1/Jaspar(0.812) More Information \| Similar Motifs Found | motif file (matrix) |
